# Plasmid-Mediated AmpC: Prevalence in Community-Acquired Isolates in Amsterdam, the Netherlands, and Risk Factors for Carriage

**DOI:** 10.1371/journal.pone.0113033

**Published:** 2015-01-14

**Authors:** E. Ascelijn Reuland, Teysir Halaby, John P. Hays, Denise M. C. de Jongh, Henrieke D. R. Snetselaar, Marte van Keulen, Petra J. M. Elders, Paul H. M. Savelkoul, Christina M. J. E. Vandenbroucke-Grauls, Nashwan al Naiemi

**Affiliations:** 1 Medical Microbiology and Infection Control, VU University Medical Center, Amsterdam, The Netherlands; 2 Laboratory for Medical Microbiology and Public Health, Hengelo, The Netherlands; 3 Department of Medical Microbiology and Infectious Diseases, Erasmus MC, Rotterdam, The Netherlands; 4 EMGO Institute for Health and Care Research, VU University Medical Center, Amsterdam, The Netherlands; 5 Medical Microbiology and Infection Control, Ziekenhuisgroep Twente, Almelo, The Netherlands; The University of Hong Kong, HONG KONG

## Abstract

**Objectives:**

The objective of this study was to determine the prevalence of pAmpC beta-lactamases in community-acquired Gram negative bacteria in the Netherlands, and to identify possible risk factors for carriage of these strains.

**Methods:**

Fecal samples were obtained from community-dwelling volunteers. Participants also returned a questionnaire for analysis of risk factors. Screening for pAmpC was performed with selective enrichment broth and a selective screening agar. Confirmation of AmpC-production was performed with two double disc combination tests: cefotaxime and ceftazidime with either boronic acid or cloxacillin as inhibitor. Multiplex PCR was used as gold standard for detection of pAmpC. 16S rRNA PCR and AFLP were performed as required, plasmids were identified by PCR-based replicon typing. Questionnaire results were analyzed with SPSS, version 20.0.

**Results:**

Fecal samples were obtained from 550 volunteers; mean age 51 years (range: 18–91), 61% were females. pAmpC was present in seven E. coli isolates (7/550, 1.3%, 0.6–2.7 95% CI): six CMY-2-like pAmpC and one DHA. ESBL-encoding genes were found in 52/550 (9.5%, 7.3–12.2 95% CI) isolates; these were predominantly blaCTX-M genes. Two isolates had both ESBL and pAmpC. Admission to a hospital in the previous year was the only risk factor we identified.

**Conclusions:**

Our data indicate that the prevalence of pAmpC in the community seems still low. However, since pAmpC-producing isolates were not identified as ESBL producers by routine algorithms, there is consistent risk that further increase of their prevalence might go undetected.

## Introduction

Resistance to broad-spectrum cephalosporins is considered to be mainly caused by extended-spectrum beta-lactamases (ESBLs). Another group of enzymes that can hydrolyze cephalosporins are the AmpC beta-lactamases. AmpC were originally described as chromosomally encoded beta-lactamases, particularly in *Enterobacter* spp., *Citrobacter freundii*, and *Serratia* spp. Plasmid-mediated AmpC (pAmpC) are AmpC beta-lactamases encoded on plasmids and hence transferable between species. These enzymes appeared in Enterobacteriaceae that lack chromosomal AmpC enzymes (*Proteus mirabilis*, *Salmonella* spp and *Klebsiella* spp) or only express low basal amounts of AmpC like *Escherichia coli* and *Shigella* spp. The frequency of pAmpC may be of larger concern than initially thought, especially if this resistance threat would mimick the trend that we have seen occurring over the past years for ESBLs [1, 2]. We consider it important therefore, to closely monitor the occurrence of this resistance trait.

Outbreaks of pAmpC have been recognized in different settings worldwide [3–8]. Currently little information is available regarding the prevalence of this group of beta-lactamases in the Dutch community. The exact prevalence of pAmpC is still unknown because simple and valid detection methods are not available, hence pAmpC-producing organisms are often missed. While algorithms for the routine detection of resistance among Gram-negative bacteria, including detection of ESBL and carbapenemases, are widely available, such algorithms are still lacking for pAmpC [9, 10].

The objective of the present study was to determine the prevalence of pAmpC beta-lactamases in community-acquired Gram negative bacteria in the Netherlands, and to identify possible risk factors for carriage of these strains.

## Materials and Methods

### Study population

In the context of a larger study aimed at determining the prevalence of carriage of ESBL-positive isolates in the community in The Netherlands, volunteers for this study were approached through five general practices, affiliated to the Academic General Practice Network, VU University Medical Center, in the region of Amsterdam. In the Netherlands, health insurance is obligatory and all inhabitants have to be registered with a general practitioner, regardless of their health status. We took advantage of this registration system, and used it to approach the study subjects. All persons older than 18 years, registered in the above mentioned five general practices were approached by postal mail, except for a small group of terminally ill patients registered with a single practitioner who preferred that these patients were not asked to participate. This means that the persons who participated in the study were not hospitalized, nor visiting their physician at that moment, hence truly recruited from the community. Volunteers were asked to send in a fecal sample and to fill in a questionnaire.

### Ethics Statement

Written informed consent was obtained from all participants, and the study was approved by the medical ethics committee (METc, NL29769.029.09) of the VU University Medical Center (NTR Trial ID NTR2453).

### Antimicrobial susceptibility testing and phenotypic confirmation of ESBL and pAmpC

Fecal samples were inoculated into Trypticase Soy enrichment Broth containing 50 mg/L ampicillin (TSB-amp) and incubated overnight at 37°C. For ESBL and AmpC screening, an aliquot of the overnight culture was subcultured on a selective screening agar which is routinely used for ESBL screening (EbSA ESBL agar, Cepheid Benelux, Apeldoorn, the Netherlands). This agar consists of a double MacConkey agar plate supplemented with vancomycin to inhibit gram-positive enterococci (64 mg/L) and cloxacillin to inhibit AmpC producers (400 mg/L) on both sides. Additionally, cefotaxime (1mg/L) was added to one of the sides, and ceftazidime (1 mg/L) to the other side to screen for isolates resistant to third generation cephalosporins. In order to detect also AmpC producers (both chromosomally and plasmid encoded AmpC), an adapted agar without cloxacillin was used in this study. Colonies growing on either side of the adapted screening agar were regarded as suspect for ESBL- and/or AmpC production and were further analyzed.

Species identification and antibiotic susceptibility testing were performed with the Vitek 2 system (bioMérieux, Marcy l’Etoile, France). The MIC breakpoints used for interpreting the results were set according to EUCAST criteria [11].

For species identification, amplification and sequencing of the 16s rRNA gene was performed on isolates for which Vitek 2 did not provide conclusive results. Phenotypic confirmation of ESBL production was performed according to the Dutch national guidelines for ESBL detection with the double disk combination test, i.e. synergy with clavulanic acid was tested for cefotaxime, ceftazidime and cefepime on Mueller-Hinton agar (Rosco, Taastrup, Denmark) [9]. In the Netherlands we assume that ESBL detection algorithms will detect all plasmid-mediated resistance to extended-spectrum cephalosporins.

According to this guideline for detection of ESBL, enterobacterial species can be divided, with regard to the presence of chromosomal AmpC which may interfere with ESBL detection, in two groups: group I comprises *Escherichia coli*, *Klebsiella* spp., *Proteus mirabilis*, *Salmonella* spp., and *Shigella* spp., in which inducible or derepressed chromosomal ampC enzyme are uncommon or absent, and group II including *Citrobacter freundii*, *Enterobacter* spp., *Hafnia alvei*, *Morganella morganii*, *Providencia* spp. and *Serratia* spp. in which the presence of inducible chromosomal AmpC beta-lactamase is more rule than exception [9]. Reduced susceptibility to cefoxitin was determined with Vitek 2 (bioMerieux, Marcy-l’Etoile, France), and was defined as an MIC > 8 mg/L according to EUCAST guidelines [11]. In the present study we included all isolates of group I and II Enterobacteriaceae, regardless of their susceptibility to cefoxitin [11]. A MIC ≤ 8 mg/L for cefoxitin was repeated with Vitek 2 and confirmed with Etest cefoxitin on Mueller-Hinton agar (bioMérieux, Solna, Sweden).

Two disk-based tests have been proposed to detect AmpC activity, namely cefotaxime and ceftazidime combined with well-known inhibitors of pAmpC: boronic acid (PBA) or cloxacillin (Rosco, Taastrup, Denmark) [12, 13]. An increase in zone diameter of ≥ 5 mm in the presence of the inhibitor indicated a positive AmpC test.

### Molecular analyses

All phenotypically confirmed AmpC and ESBL positive isolates were analyzed by PCR for molecular detection of pAmpC genes. Bacterial DNA was isolated using the QIAamp DNA mini kit (QIAGEN, Venlo, the Netherlands), and an initial multiplex screening PCR was performed, followed by a confirmatory singleplex PCR for multiplex PCR positive DNA. The primers used were specific for MOX-type, CMY-type, DHA-type, ACC-type, MIR-/ACT-type and FOX-type, with a slightly increased primer annealing step of 70°C for 30 seconds [14]. Detection by PCR was considered the gold standard for pAmpC detection. Group I isolates were also screened for AmpC-encoding genes by Check-MDR CT103 microarray to identify CMY I/MOX, CMY II, FOX, DHA, ACT/MIR and ACC (Check-Points Health BV, Wageningen, the Netherlands) [15]. In isolates positive for ESBL in the phenotypic tests, ESBL genes were characterized by PCR (VUmc) and sequencing (BaseClear, Leiden, the Netherlands) [16, 17].

### Identification of plasmids and epidemiological typing

Characterization of enterobacterial plasmids was performed with PCR-based replicon typing for the detection of the eight most prevalent replicon types [18]. Seven AmpC-positive *E. coli* isolates were analyzed for genetic relatedness by Amplified-fragment length polymorphism (AFLP), as described [19]. Bionumerics software, version 6.6, (Applied Maths, Sint-Martens-Latem, Belgium) was used to analyze AFLP banding patterns.

### Statistical analyses

Statistical analyses were performed with SPSS, version 20.0.

## Results

Between August 12 and December 13, 2011, fecal samples and questionnaires were obtained from 550 volunteers. The mean age of participants was 51 years (range: 18–91), and 61% of participants were female.

With the phenotypic AmpC confirmation test, (AmpC disk diffusion combination test with boronic acid and cloxacillin), we detected 176/550 (32%) AmpC positive isolates; these included Enterobacteriaceae from both Group I and Group II. Among group I species, 45/176 isolates (*42 Escherichia coli*, 2 *Klebsiella pneumoniae*, 1 *Salmonella* spp) were positive in the phenotypic confirmation test. Only 7/42 of the *E. coli* isolates, however, were confirmed to be positive for pAmpC by multiplex AmpC PCR. Among Group II species, which consisted of 60 *Enterobacter* spp, 47 *Citrobacter* spp, 16 *Hafnia alvei*, 5 *Morganella morganii*, 1 *Raoulthella ornithinolytica*, 1 *Aeromonas hydrophila/caviae*, 1 *Serratia plymuthica*, 131 isolates were found to be positive using the phenotypic confirmation test, whereas only 52 were found to be positive using the multiplex AmpC screening PCR.

If an AmpC gene was detected by PCR that is specific for the species concerned, it was considered as chromosomal [1]. The AmpC genes detected in group II species were all species-related chromosomal genes: CMY-2-like pAmpC was found in *Citrobacter freundii,* DHA in *Morganella morganii* spp., ACC in *Hafnia alvei,* and ACT/MIR-1 in *Enterobacter* species. A non-species related pAmpC was detected by PCR in 7 strains, all *E. coli* (7/550, 1.3%, 0.6–2.7 95% CI). The genes were 6 *bla*_CMY-2-like_ and 1 *bla*_DHA_. Microarray results confirmed the results obtained by PCR in group I Enterobacteriaceae [20].

ESBL-encoding genes were detected in 52/550 samples (9.5%, 7.3–12.2 95% CI), these were predominantly *bla*_CTX-M_ genes. Interestingly, two pAmpC-producing isolates also produced an ESBL: one CTX-M-1 and one CTX-M-15. Two of the seven pAmpC positive *E. coli* isolates were also resistant to cotrimoxazole, one to ciprofloxacin and one to gentamicin. Multiresistance, i.e. resistance to at least one antimicrobial agent from three or more antimicrobial categories (aminoglycosides, quinolones and cotrimoxazole), was detected in a single isolate [21]. One isolate was also resistant to nitrofurantoin. All pAmpC-producing strains were susceptible to meropenem and imipenem. No cefoxitin susceptible (as determined by E-test) pAmpC-positive isolates were found.

AFLP was performed on the pAmpC-producing *E. coli* strains, which showed that the seven isolates were not genetically related. In these seven pAmpC-producing isolates, the plasmid replicon types IncI1 (5/7), ColE (5/7), ColEtp (3/7), FIB (3/7), Frep (2/7), R (1/7) and FIA (1/7) were identified. We did not perform transformation experiments to precisely identify the pAmpC-carrying plasmid types. The types of plasmids we detected, however, are in accordance with previous publications that show that a wide range of plasmid replicon types may be associated with pAmpC-positive bacterial isolates, including A/C, I1, Y, F, K, FII, L/M and B/O [22–24].

After analysis of the 544 questionnaires (six participants did not complete the questionnaire), our data showed that all the seven cases were women, with four out of six aged between 18 and 30 years (Table 1). No comorbidities were present. Only two out of seven cases had used antibiotics in the previous year (tetracyclines seven months and quinolones 12 months earlier), compared to 80 out of 495 persons who did not carry pAmpC (OR 2.6, CI 0.5–14.4). Three out of seven cases were admitted to a hospital in the Netherlands (OR 7.2; CI 1.6–33.2), one had an admission to a foreign hospital and one participant was admitted to a rehabilitation center. Healthcare-associated pAmpC carriage overall turned out to be significant (OR 6.9; CI 1.5–31.5). Four carriers visited countries inside Europe, only one travelled to Asia (5 months ago) and one to Africa (more than one month ago).

**Table 1:** Characteristics of the participants (n = 544) included.

	**pAmpC carrier (7)**	**pAmpC non-carrier (537)**	**total**	**OR**	**CI**
18–30 years	4/6	148/537	544	5.4	1.0–9.5
Previous use of antibiotics	2/7	80/495	501	2.6	0.5–14.4
Healthcare-associated*	3/7	52/528	535	6.9	1.5–1.5
Travel outside Europe	2/7	192/533	540	0.7	0.1–3.7

*Healthcare-associated acquisition included hospital admission in the Netherlands or in a foreign country, or admission to a rehabilitation center.

## Discussion

In this study we detected pAmpC-positive enterobacterial isolates in seven out of 550 (1.3%) fecal samples obtained from community-dwelling individuals in the region of Amsterdam. The participants in the study were approached by postal mail, hence they were not hospitalized, nor attending their physician at the moment of recruitment. Because of the unbiased way we approached the study population, participants represent a cross-section of the general population (older than 18 years) and therefore include healthy persons, and persons that may have been in hospital before or may have recently visited their general practitioner for any reason. We consider therefore the prevalence that we measured to reflect the actual prevalence of carriage of pAmpC in the general population.

To date, pAmpC has been found mainly in clinical isolates obtained from hospitalized patients [25, 26]. Only very limited data are available regarding the prevalence of pAmpC circulating in the community [2]. Data vary from 0.59% in outpatients in Spain to 6.7% in Libya and 16% in another study performed in Spain.[2, 27–29] More than one percent in the present study seems however to be high in strains isolated from community patients in a country with prudent antibiotic use. The possible relevance of community circulating pAmpC has been previously indicated by Pitout *et al*., who showed that pAmpC-producing Enterobacteriaceae are relevant community pathogens with important implications for public health, especially as a cause of urinary tract infections in older women [30]. In addition, studies from different countries show that the community serves as a reservoir for the introduction of pAmpC in the hospital setting [31, 32].

A few studies analyzed possible risk factors for infections due to pAmpC and ESBL-producers. Our results should be interpreted with caution, because of the very small number of cases, but the possible risk factors that we found are similar to those described previously, i.e. contact with healthcare [1, 2]. It is interesting that, despite the small number of carriers we found, the association with female gender is apparent and comparable to that found by Pitout (although there is a difference in age) [30].

All seven pAmpC-producing Enterobacteriaceae we found were E. coli isolates, a species which is also the predominant ESBL-producing species among community-acquired ESBL-producers [33, 34]. The most frequent pAmpC gene belonged to CMY group II; this is comparable to what has been found in several previous studies, where *bla*_CMY-2_ was found to be the most widely distributed pAmpC gene geographically [30, 31, 35].

pAmpC-encoding genes are often located on large plasmids, which are associated with multidrug resistance [36]. Two of the seven pAmpC-positive *E. coli* strains that we identified in the present study also harboured an ESBL gene. These strains were therefore resistant to cephalosporin antibiotics by multiple mechanisms and could easily be detected by routine diagnostic methods. Five of the seven pAmpC-producing isolates, however, were phenotypically ESBL negative, meaning that these strains would be missed as strains possessing a plasmid-mediated resistance to extended-spectrum cephalosporins. Hence, in the routine setting, the prevalence of pAmpC-producing strains is probably largely underestimated. Phenotypic detection of pAmpC has such poor specificity that it cannot be used for routine pAmpC detection. The only reliable pAmpC detection methods are multiplex PCR and specific microarrays such as the Check-MDR CT103 [20]. These molecular methods however, are only applicable to species of Group I Enterobacteriaceae, since in Group II Enterobacteriaceae isolates, a positive pAmpC PCR result is most likely due to the presence of chromosomal AmpC genes. Indeed, plasmid-encoded genes can be identical to chromosomally located AmpC beta-lactamase genes in this group of Enterobacteriaceae [1].

Several of the pAmpC-producing strains were also resistant to aminoglycosides, quinolones, cotrimoxazol and nitrofurantoin. High rates of co-resistance have also been reported in other studies, which means that also for infections caused by pAmpC-producing strains there may be few therapeutic options. This could increase morbidity and mortality in affected patients [2, 37, 38].

In conclusion, a pAmpC prevalence of 1.3% (all *E. coli*) was observed in a Dutch community setting. Importantly, the majority of the pAmpC-producing isolates were not detected by routine phenotypic screening algorithms for ESBLs. Although plasmid-mediated ESBL production seems to spread much more easily than plasmid-mediated AmpC production, careful monitoring of this plasmid-mediated resistance mechanism seems appropriate.

## Supporting Information

S1 Table (SAV)
